# PILOTING THE MONEY SMART FINANCIAL LITERACY PROGRAM IN JAMAICA

**DOI:** 10.1093/geroni/igz038.2153

**Published:** 2019-11-08

**Authors:** Douladel Willie-Tyndale

**Affiliations:** Mona Ageing & Wellness Centre, Kingston, Jamaica

## Abstract

This paper describes the piloting of a culturally modified version of the FDIC Money Smart Peer-to-Peer Program. The pilot study examined the use of Mentorship Teams comprised of two persons one (60 years and over) and another (30 - 59 years) who collectively provided mentorship and financial education to four working-age adults. The pilot was designed with four (4) phases: Phase 1 included recruitment and training of Mentors, Phase 2 the assignment of Mentors and Mentees, Phase 3 application of the intervention, and Phase 4 the evaluation of outcomes. This paper will review the design and recruitment processes, modifications made to the existing Money Smart Peer-to-Peer Program for cultural sensitivity and financial system factors, implementation challenges (including barriers and facilitators) and lessons derived from initiation and program implementation.

